# Suspended Materials in River Waters Differentially Enrich Class 1 Integron- and IncP-1 Plasmid-Carrying Bacteria in Sediments

**DOI:** 10.3389/fmicb.2018.01443

**Published:** 2018-07-02

**Authors:** Magali De la Cruz Barrón, Christophe Merlin, Hélène Guilloteau, Emmanuelle Montargès-Pelletier, Xavier Bellanger

**Affiliations:** ^1^LCPME, CNRS, Université de Lorraine, Nancy, France; ^2^LIEC, CNRS, Université de Lorraine, Nancy, France; ^3^LTSER France, Zone Atelier du Bassin de la Moselle, Nancy, France

**Keywords:** class 1 integrons, IncP-1 plasmids, environmental reservoirs of antibiotic resistance genes, river water column, suspended matter, sediments, biofilm, horizontal gene transfer

## Abstract

Aquatic ecosystems are frequently considered as the final receiving environments of anthropogenic pollutants such as pharmaceutical residues or antibiotic resistant bacteria, and as a consequence tend to form reservoirs of antibiotic resistance genes. Considering the global threat posed by the antibiotic resistance, the mechanisms involved in both the formation of such reservoirs and their remobilization are a concern of prime importance. Antibiotic resistance genes are strongly associated with mobile genetic elements that are directly involved in their dissemination. Most mobile genetic element-mediated gene transfers involve replicative mechanisms and, as such, localized gene transfers should participate in the local increase in resistance gene abundance. Additionally, the carriage of conjugative mobile elements encoding cell appendages acting as adhesins has already been demonstrated to increase biofilm-forming capability of bacteria and, therefore, should also contribute to their selective enrichment on surfaces. In the present study, we investigated the occurrence of two families of mobile genetic elements, IncP-1 plasmids and class 1 integrons, in the water column and bank sediments of the Orne River, in France. We show that these mobile elements, especially IncP-1 plasmids, are enriched in the bacteria attached on the suspended matters in the river waters, and that a similar abundance is found in freshly deposited sediments. Using the IncP-1 plasmid pB10 as a model, *in vitro* experiments demonstrated that local enrichment of plasmid-bearing bacteria on artificial surfaces mainly resulted from an increase in bacterial adhesion properties conferred by the plasmid rather than an improved dissemination frequency of the plasmid between surface-attached bacteria. We propose plasmid-mediated adhesion to particles to be one of the main contributors in the formation of mobile genetic element-reservoirs in sediments, with adhesion to suspended matter working as a selective enrichment process of antibiotic resistant genes and bacteria.

## Introduction

Predictions of future scenarios regarding the consequences associated with the burden of antibiotic resistant bacteria (ARB) in the forthcoming years are still a matter of debate (de Kraker et al., 2016), but there is no doubt that this burden is already impairing our ability to treat common infectious diseases (Cosgrove, 2006). Natural environments, especially soils and aquatic ecosystems, are believed to play a key role in the emergence and dissemination of ARB. Indeed, being the final recipient of wastewater discharges or solid wastes from animal husbandry, such environments are often exposed to anthropogenic chemical pollutants such as antibiotics and biocides, as well as microbial pollutants such as ARB (Baquero et al., 2008; Knapp et al., 2010; Heuer et al., 2011). Considering the ever-increasing occurrence of ARB in the anthropogenic-impacted environments, these bacteria and their antibiotic resistance genes (ARGs) are now considered as environmental contaminants of emerging concern (Pruden et al., 2006).

Several studies have shown that, globally, the environment can act as a reservoir of ARGs and that many Gram-positive and Gram-negative pathogens have acquired new resistance traits originating from this huge gene pool (Martinez, 2009; Wright, 2010; Perry and Wright, 2013). Part of the problem arises from the association of ARGs with so-called mobile genetic elements (MGEs) that allow them to be horizontally transferred between environmental and/or clinical bacteria. MGEs represent a rather large variety of elements including conjugative plasmids, integrative and conjugative elements, transposons, and integrons (Frost et al., 2005), some of them being more often associated with ARGs than others. As a consequence, instead of monitoring the occurrence of a large panel of clinically relevant ARGs, some authors prefer focusing on selected MGEs frequently associated with ARGs. Among them, class 1 integrons, and to a lesser extent, IncP-1 conjugative plasmids, have been widely studied as proxies to assess the global ARG content of environmental matrices (Stalder et al., 2012; Rizzo et al., 2013; Gillings et al., 2015; Jechalke et al., 2015). Class 1 integrons are genetic platforms able to capture and express resistance gene cassettes, thus promoting genotypic/phenotypic diversity and adaptation of bacteria (Mazel, 2006). They are minimally constituted of an *intI1* gene encoding a site-specific recombinase and of a recombination site *attI* where IntI1 catalyzes the gene cassette insertions. Class 1 integrons usually carry 1–4 of more than 130 different resistance gene cassettes that have been described to date, which makes them integrated indicators of ARGs (Partridge et al., 2009; Stalder et al., 2014; Cury et al., 2016). The plasmids of the IncP-1 incompatibility group are broad host range conjugative elements (Adamczyk and Jagura-Burdzy, 2003). These plasmids, found in clinical and environmental contexts, often carry multiple antibiotic resistance determinants suggesting that they also play a significant role in ARG dissemination (Popowska and Krawczyk-Balska, 2013).

It has been demonstrated that ARGs tend to accumulate over time in environmental microbial communities as a probable consequence of anthropogenic pressure (Knapp et al., 2010). Their association with MGEs can explain the persistence of ARGs in the environment as MGEs allow them to be transferred from unfit bacteria originating from the animal/human microbiome to more locally adapted bacteria (Frost et al., 2005; Martinez, 2009). Surprisingly, MGEs are not equally distributed in heterogeneous matrices, with specific enrichment of some MGEs on suspended particles in marine waters, as demonstrated by Ganesh and collaborators (Ganesh et al., 2014). This suggests that MGE-bearing bacteria can selectively and locally be enriched. The reason for such local and selective enrichment of MGEs is not fully elucidated but it has been known for some time that, besides their ability to transfer genes, MGEs, such as conjugative plasmids, also influence surface properties of bacterial cells (Van Houdt and Michiels, 2005). Indeed, the conjugation of plasmids implies the synthesis of external cell appendages (conjugative pili or adhesins in either Gram-negative or Gram-positive bacteria) that have been demonstrated *in vitro* to promote cell adhesion to solid surface and biofilm formation (Ghigo, 2001; Bhatty et al., 2015, 2017). On the principle, bacterial adhesion thanks to plasmid pili and conjugative transfer of plasmids are two phenomena that might contribute to the formation of local reservoirs of enriched plasmid occurrence, but their relative contributions in a context as complex as a river remain to be elucidated.

In the present work, we investigated the relative occurrence of two MGE proxies for ARGs, namely class 1 integrons and IncP-1 plasmids, in the water column and surface sediments of the Orne River (France) by analyzing raw waters, suspended materials (SMs), and sediments. The results obtained tend to show that bacteria carrying IncP-1 plasmids, and to a lesser extent class 1 integrons, selectively attached on SMs that finally settle to form sediments. *In vitro* experiments were further used to assess the contribution of IncP-1 plasmids to the binding of plasmid-bearing bacteria to artificial supports, and the contribution of bacterial adhesion to plasmid dissemination. All in all, our results demonstrate the major contribution of plasmid-mediated adhesion to particles to the formation of MGE reservoirs in sediments.

## Material and Methods

### Bacterial Strains and Growth Conditions

The bacterial strains and plasmids used in this work are presented in **Table 1**. Bacteria were grown aerobically in LB medium (LB Broth Miller, Difco^TM^) at 30°C, with agitation at 160 rpm for liquid cultures. Solid medium was prepared by adding 15 g/L of agar. When required, antibiotic selection was applied at the following concentrations: ampicillin at 100 mg/L, rifampicin at 20 mg/L, and tetracycline at 10 mg/L.

**Table 1:** Bacteria and plasmids used in this study.

Name/species	Genotype/characteristics^a^	References
***Plasmids***		
pB10	Wild type IncP-1 plasmid isolated from activated sludge	Schlüter et al. (2003)
pBELX	pEX-A derivative (pUC18-based) containing qPCR target sequences for *trfA* of IncP-1 plasmids, the Eubacterial 16S rRNA gene	Bellanger et al. (2014b)
pNORM1	pEX-A derivative (pUC18-based) containing the qPCR target sequences for *intI* of class 1 integron, the Eubacterial 16S rRNA gene	Gat et al. (2017)
***Bacillus subtilis***		
LMG 7135^T^	ATCC 6051, Type strain	BCCM/LMG^b^
CM291	Rif^R^ derivative of LMG 7135^T^	This work
CM295	CM291(pB10), Rif^R^, Amx^R^, Str^R^, Sul^R^, Tet^R^	This work
***Cupriavidus metallidurans***		
AE815	plasmid free and Rif^R^ derivative of wild type strain CH34	Springael et al. (1993)
CM124	AE815(pB10), Rif^R^, Amx^R^, Str^R^, Sul^R^, Tet^R^	Bellanger et al. (2014a)
***Delftia acidovorans***		
CM122	*Delftia acidovorans*, Rif^R^, Kan^R^	Eva Top lab strain collection
CM294	CM122(pB10), Rif^R^, Kan^R^, Amx^R^, Str^R^, Sul^R^, Tet^R^	This work
***Escherichia coli***		
DH5α	ϕ80*lacZ*ΔacZe*recA1endA1gyrA96* (Nal^R^) *thi-1 hsdR17(r_K_^-^m_K_^+^) supE44 relA1 deoR Δ(lacZYAargF) U169*	Sambrook et al. (1989)
MG1655	Sequenced λ^-^ and F^-^derivative of strain K-12	Blattner et al. (1997)
CM102	DH5α(pB10), Nal^R^, Amx^R^, Str^R^, Sul^R^, Tet^R^	Schlüter et al. (2003)
CM125	Nal^R^ derivative of MG1655	Bellanger et al. (2014a)
CM278	CM125(pB10), Nal^R^, Amx^R^, Str^R^, Sul^R^, Tet^R^	This work
***Pseudomonas fluorescens***		
CIP 69.13^T^	ATCC 13525, Type strain	ATCC^c^
CM292	Rif^R^ derivative of wild type strain CIP 69.13^T^	This work
CM297	CM292(pB10), Rif^R^, Amx^R^, Str^R^, Sul^R^, Tet^R^	This work
***Pseudomonas putida***		
SM1443	KT2442 (Rif^R^) with a mini-Tn*5-lacI^q^* insertion	Christensen et al. (1998)
CM236	SM1443(pB10), Rif^R^, Amx^R^, Str^R^, Sul^R^, Tet^R^	This work
***Shewanella oneidensis***		
CM87	Rif^R^ derivative of wild type strain MR-1^T^	Schwalb et al. (2003)
CM293	CM87(pB10), Rif^R^, Amx^R^, Str^R^, Sul^R^, Tet^R^	This work


*^*a*^Antibiotic resistance phenotype: Amx^*R*^, amoxicillin; Kan^*R*^, kanamycin; Nal^*R*^, nalidixic acid; Rif^*R*^, rifampicin; Str^*R*^, streptomycin; Sul^*R*^, sulfamides; Tet^*R*^, tetracycline. ^*b*^Belgian coordinated collections of microorganisms. ^*c*^American Type Culture Collection.*

### Study Site and River Material Sampling

The Orne is an 86 km long river flowing in Lorraine, tributary of the Moselle in northeastern France, and sub-tributary of the Rhine. The Orne is one of the rivers of the area strongly impacted by ancient mining and iron- and steel-making plants during the 20th century. The Orne River takes its source in a forest area, flows through countryside consisting of fields and meadows, then a highly urbanized and ancient industrialized area before joining the Moselle River. The latter part represents about one fourth of the river linear and, based on geographical data, it is assumed that the river waters drain between 86 and 100% of the watershed surface (1276 km^2^), depending on the sampling site considered in this study (see Supplementary Figure 1). The riverbed was strongly modified for industrial purposes. Indeed, two dams were built in the 1958–1965 period and the river was calibrated and channeled in the very last kilometers (Abuhelou et al., 2017; Kanbar et al., 2017). Fifteen pairs of river raw water and SM samples were collected along the last 23 km of the Orne River and over an 11-month period from November 2014 to October 2015 (four pairs on 2014/11/04, three pairs on 2015/02/02, two pairs on 2015/02/03, three pairs on 2015/05/05, and three pairs on 2015/10/06). As previously described (Le Meur et al., 2016), the river water was pumped and sent to a continuous flow field CEPA Z-41 centrifuge (20,000 RPM, equivalent to 17,000 × *g*, with a 600 L/h flow rate), which was used to collect a representative SM-enriched fraction from [*ca*.] 1–2 m^3^ of raw water. Raw water samples were collected from the pumping outlet (excess flow). The top surface sediments were collected along the same Orne River section at five different places from January 2014 to July 2015. Sediment samples were collected (on 2014/03/18 and 2015/02/19) as short cores using a piston corer or simple coring tubes (diameter of 6 cm or 9 cm). Coring was preferred to grabbing in order to preserve the layered structure of sediments. The collected sediments were sealed from air and transported in a vertical position. The upper 2 cm layer, referred to as surface sediments, was separated from the core using a single use spatula and into an N_2_-filled glove bag to avoid oxidation. The surface layer is highly hydrated, and loosely attached to the underneath material. Different aliquots were prepared for distinct purposes. For geochemical analyses, an aliquot was freeze-dried and ground using an agate mortar and pestle. For DNA extraction, all samples were kept frozen at -20°C (short term storage of SMs and raw waters) or -80°C (long term storage of sediments).

### Geochemical Analyses on Sediments and Suspended Materials

Major elements were detected by inductively coupled plasma optical emission spectrometry (ICP-OES). These analyses were performed at SARM (Service d’Analyse des Roches et des Minéraux – CRPG, Vandœuvre-lès-Nancy, France) and all analytical methods were subject to QC/QA procedures using certified reference materials (Carignan et al., 2001). Grain size distribution of SMs and bottom sediments was obtained using laser diffraction (SYMPATEC) with two distinct lenses corresponding to two distinct size ranges (0.45–87.5 μm and 4.5–875 μm). Samples were systematically ultrasonicated for 20 s before measurement, and each measurement was duplicated or triplicated. Mineralogy of sediment and SM samples was investigated using X-ray Diffraction and Transmission Electron Microscopy (like in Kanbar et al., 2017). A synthesis of those analyses is available in Supplementary Table 1 and Supplementary Figures 2, 3.

### DNA Extraction

Recombinant plasmid DNAs pBELX and pNORM1, used as qPCR standards, were extracted using the “Wizard^®^ Plus SV Minipreps DNA Purification System” (Promega) according to the recommendations provided by the manufacturer. The plasmids were linearized by *Bam*HI (Promega) before being purified with the “QIAquick PCR DNA purification kit” (Qiagen). Total environmental DNAs were extracted using the “PowerWater DNA Isolation Kit” (MO BIO laboratories Inc). Briefly, 50 mg of sediments or SMs were thawed before being dispersed in 100 mL of non-pyrogenic sterile water (Aqua B-Braun) by vortexing for 30 s followed by 15 min stirring at 160 rpm and 25°C. These sediment/SM suspensions (or 100 mL of sample for raw waters) were filtered on polycarbonate filters (Whatman Nuclepore filter, pore size 0.22 μm, diameter 47 mm) using a filtration apparatus (Combisart 6-branch Manifold, Sartorius). Total DNAs were directly extracted from the filters according to the recommendations provided by the manufacturer and were eluted from silica columns with 100 μL of PCR grade water (RNase-Free Water, Qiagen). Plasmid and total DNA concentration and purity were estimated by spectrophotometry according to standard procedures, and all DNAs were stored at -20°C until use.

### Quantitative PCR Assays

The abundance of class 1 integrons and IncP-1α/β plasmids were quantified by qPCR in total environmental DNAs using “Power SYBR^®^ Green PCR Master Mix” (Applied Biosystems) with the primers listed in **Table 2**. Quantitative PCRs were performed in triplicate using “Step One Plus Real-Time PCR System” (Applied Biosystems, driver: StepOne Software v2.2) in a 25 μL reaction volume with 1 μM of each primer, and with thermocycling conditions set as follows: 10 min at 95°C followed by 45 cycles of 15 s at 95°C and 1 min at 60°C. The quality of the PCR products was subsequently checked by melting curve analyses, for which the temperature was ramped between 60 and 95°C in increments of 0.3°C. IncP-1α/β plasmid and class 1 integron quantitative results were normalized to the amount of eubacterial 16S rRNA gene, also quantified by qPCR with the 331F/518R universal primers and using the cycling conditions described above. For quantifications of 16S rRNA gene and IncP-1α/β plasmids and of class 1 integrons, plasmids pBELX and pNORM1, linearized with *Bam*HI, were used as standards, respectively (**Table 1**). The absence of residual inhibitors in the DNA extracts was checked by qPCR by comparing amplifications from serially diluted DNA templates.

**Table 2:** Primers used in qPCR.

Name	Sequence	Targeted gene	Product size (bp)	Referencec
331F	5′-TCCTACGGGAGGCAGCAGT-3′	16S rRNA	197 bp	Muyzer et al., 1993; Nadkarni et al., 2002
518R	5′-ATTACCGCGGCTGCTGG-3′			
intI1-LC1	5′-GCCTTGATGTTACCCGAGAG-3′	*intI1* (class 1 integrons)	196 bp	Barraud et al., 2010
intI1-LC5	5′-GATCGGTCGAATGCGTGT-3′			
trfA2-1	5′-CGAAATTCRTRTGGGAGAAGTA-3′	*trfA* (IncP-1 plasmids)	241 bp	Götz et al., 1996
trfA2-2	5′-CGYTTGCAATGCACCAGGTC-3′			


### Crystal-Violet Staining of Adhering Biomass

Two milliliters of LB was inoculated with a single colony picked from a fresh plate, containing tetracycline for pB10-carrying bacteria, and then incubated for 16 h. One hundred microliters of this culture was mixed with 10 mL of fresh medium and placed in a 55 mm petri dish containing a 47 mm-diameter flat polyethylene disk (Kaldnes Biochip Media) that are originally dedicated to the colonization of bacteria in moving bed biofilm reactors in wastewater treatment processes. The plates were incubated at 30°C under agitation (80 rpm) for 18 h. The quantity of biomass adhering to Kaldnes polyethylene disks was determined by classical crystal-violet staining (Chavant et al., 2007). First, the total amount of planktonic bacteria surrounding the polyethylene disks was estimated by measuring broth turbidity (OD_600nm_). Second, the polyethylene disks were transferred into new petri dishes where they were successively washed three times with 10 mL of sterile water for removing poorly adherent bacteria. The polyethylene disks were then air-dried for 1.5 h before being incubated in 10 mL of a 0.1% v/v crystal violet solution in H_2_O (Biomérieux) for 45 min at room temperature (20°C ± 1°C). The stained disks were then gently washed six times with sterile water to remove excess of crystal violet. Finally, the biomass-associated crystal violet was dissolved with 10 mL of glacial acetic acid and quantified by UV-Vis spectrophotometry at 540 nm (OD_540nm_). Polyethylene disks without exposure to bacterial cells were used as control.

### Bacterial Mating Assays

Mating assays were performed in liquid medium either containing or not a 47 mm polyethylene disk (Kaldnes Biochip Media). Donor (CM102) and recipient bacteria (AE815) were grown for 16 h in broth, supplemented or not with antibiotics, then washed by centrifugation and re-suspended in one volume of sterile MgSO_4_ (10 mM). For mating assays with a polyethylene disk, 10 mL of LB broth containing a 1:100 dilution of both strains, prepared from the washed cell suspensions, were introduced in an empty 55 mm petri dish and incubated for 18 h at 30°C under agitation (80 rpm). At the end of the incubation period, broth and polyethylene disks were recovered separately for bacterial strain enumeration. Beforehand, the polyethylene disks were washed three times with sterile water and adhering bacteria were re-suspended by vigorous vortexing in 20 mL of MgSO_4_ (10 mM). Donor (CM102), recipient (AE815), and transconjugant bacteria [AE815(pB10)] were enumerated on selective plates containing either tetracycline (Tet), rifampicin (Rif), or both antibiotics (Tet Rif), respectively. For the control mating assays between planktonic cells, the incubations of donor and recipient cells were performed as described above but the polyethylene disks were omitted.

### Statistical Analyses

Statistical analyses were performed using the R software and presented according to Cumming and collaborators (Cumming et al., 2007). The normality of the data was verified using the Shapiro-Wilks test before performing other statistical tests. The potential pairing between data was also taken into account before carrying out the analyses. For statistical analyses of the data, Wilcoxon signed-rank test, Wilcoxon rank-sum test, Kruskal–Wallis rank-sum test, Student’s *T*-test, and Spearman correlation test were independently chosen according to the circumstances, as justified in the corresponding Result subsections.

## Results

### Occurrence of Class 1 Integrons and IncP-1 Plasmids in the Orne River

**FIGURE 1:**
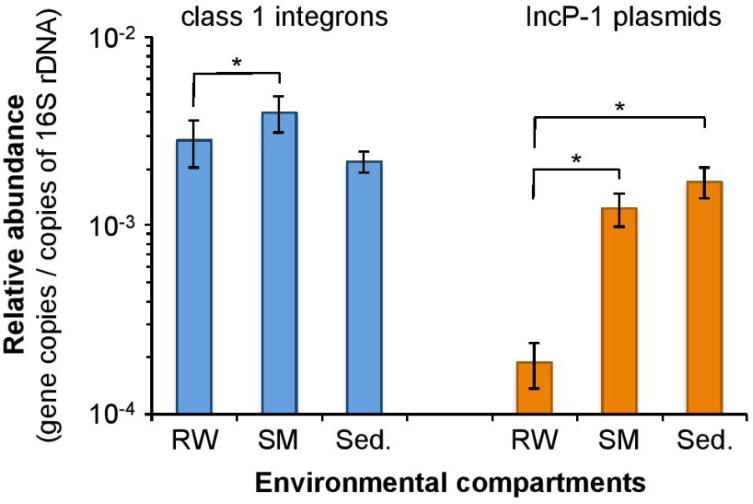
Relative abundance of class 1 integrons and IncP-1α/β plasmids in compartments from the Orne River. RW, Raw waters; SM, Suspended materials; Sed., Sediments. Error bars indicate the standard error of the mean (*n* = 15 for RW and SM; *n* = 8 for Sed.). Asterisks indicate statistical differences between relative abundances (*p* < 10^-3^).

The distribution of class 1 integrons and IncP-1α/β plasmids in the different physical compartments of the Orne River was first investigated so as to point out any selective/specific partitioning. Total community DNA was extracted from 15 raw water samples, 15 corresponding SM samples, and eight surface sediment samples collected over an 11-month period. Their contents in class 1 integrons and IncP-1α/β plasmids were determined by qPCR and then further normalized to the corresponding 16S rRNA gene content, also determined by qPCR. The relative abundance of each MGE in the three compartments is presented in **Figure 1**.

Globally, the relative abundance of class 1 integrons and IncP-1α/β appeared to be 2.2 ± 0.4 and 13.3 ± 4.0 times higher in SMs than in the corresponding raw water samples, respectively. This trend was observed in all but 1 of the 15 paired samples. Wilcoxon signed-rank test further confirmed the statistical significance of the MGE enrichment on SM-associated microbial communities with *p*-values of 3.10^-4^ and 6.10^-5^ for class 1 integrons and IncP-1α/β plasmids, respectively. Despite being both significant, the enrichment effect observed for MGE-bearing bacteria on SMs remains higher for IncP-1α/β plasmids than for class-1 integrons (Wilcoxon signed rank test; *p* = 2.10^-4^), which already points out two different bacterial populations with different MGE content characteristics.

The statistical comparison of the MGE abundances obtained for top surface sediments with those obtained for the other compartments requires using a Wilcoxon rank-sum test as sediments were sampled independently from raw waters and SMs, and data were not normally distributed. In this respect, the relative abundance of class 1 integrons in surface sediments did not seem to differ significantly from those of raw waters or SMs (*p* = 0.39 and *p* = 1, respectively). For IncP-1α/β plasmids though, the relative abundance was 9.1 and 1.4 higher in sediments than in raw waters and SMs, respectively. If these relative abundances were statistically different between sediments and raw waters (*p* = 5.10^-4^), they remained non-significantly different between surface sediments and SMs (*p* = 0.18). The latter result raises the question of whether plasmid-bearing bacteria from raw waters could selectively be enriched on SMs, which could then settle in the riverbed to form fresh sediments that exhibit a similar IncP-1 plasmid richness, as we shall discuss in subsection 3.2.

The different distributions observed between IncP-1 plasmid- and class 1 integron-bearing bacteria show that MGEs influence their own compartmentalization in aqueous media. Two hypotheses can be made to explain the specific enrichment of IncP-1 plasmids on SMs. First, IncP-1 plasmids are conjugative elements for which it has been suggested that the promiscuity encountered in biofilms favors gene transfer compared to the planktonic bacteria life style (Molin and Tolker-Nielsen, 2003). Considering the fact that conjugative transfer is a replicative event (Lanka and Wilkins, 1995), it can be argued that bacterial adhesion to SMs leads to biofilm formation, which then favors plasmid transfer and, therefore, results in an increase in its relative abundance. Alternatively, Ghigo (2001) showed that conjugative plasmids themselves could promote the formation of biofilms as they encode for a conjugative pili, a cell appendage also considered as an adhesion substrate. In such a case, the IncP-1 plasmid-bearing fraction of the bacterial population should be more likely to bind to the organo-mineral particles constituting SMs than the plasmid-free bacteria, which should lead to a compartmentalization of the plasmid-bearing bacteria over time. The two hypotheses, “adhesion-promoted plasmid transfer” and “plasmid-promoted adhesion” are not exclusive, but their relative contributions remain to be elucidated.

### Mineral Relationship Between Sediments and SMs of the Orne River

If IncP-1 plasmid-bearing bacteria were to be selectively enriched on SMs before particles settle and form sediments, a parent-like relationship between SMs and surface sediments is to be expected. In such a case, SMs and top surface sediments should share common mineral properties demonstrating their relationship in terms of size distribution, element composition, and mineral constituents (Supplementary Table 1 and Supplementary Figures 2, 3). SMs and sediments are predominated by silicates, in particular clay minerals. SM particles are relatively fine with a grain size distribution centered below 10 μm, and the decile (D50) of Orne River suspended particles was estimated to be 12 ± 3 μm. The grain size distribution measurements evidenced that sediments are coarser than SMs, suggesting a size sorting upon settling and/or a post-settlement modification of the grain size distribution, as reported elsewhere (Lartiges et al., 2001; Droppo et al., 2009). This modification can be linked to a slightly higher carbon content in sediments (4.3 ± 2% and 4.9 ± 0.7% for SMs and sediments, respectively) as organic matter is known to enhance particle aggregation. All in all, the element composition and the mineralogy clearly draw a parent-like relationship between SMs and surface sediments. Although slight differences remain, they can be attributed to both successive settling events and post-settlement modifications. Yet, a parental-like relationship between SMs and sediments can be interpreted as particle settling, as sediment mobilization, or as both.

Due the locations of the sampling stations (Supplementary Figure 1), the SMs transported by the Orne River are likely to be alternatively predominated by runoff-generated particles during rain events (or high water discharge periods), and by urban inputs and primary production in the water column during low-water discharge periods. The relative contribution of sediment remobilization as SMs is driven by different parameters including hydroclimatic conditions and river characteristics (class, width, flood bed status, sediment flow rate, etc.). This contribution is considered to be significant during specific periods of hydrological sequences, i.e., during the early stages of flood events occurring after a long low-flow period (typically during the fall season). In the studied case, most of the samples were collected in the middle or late stages of flood events (see Supplementary Figure 1), thus it can be considered that the origin of SMs sampled in the water column was predominated by particles resulting from soil leaching. In the Orne watershed, 67% of the surface is dedicated to agriculture, enhancing the generation of suspended particles during rain events (Le Meur et al., 2016). Thus, although we cannot fully demonstrate the absence of sediment remobilization, its contribution is considered to be minor in such water discharge conditions. Mineralogy, particle size distribution, and element contents clearly draw a parental-like link between sediments and SMs. Thus, these similarities are explained by the SM settling process, which mostly occurs at the end of the flood events, i.e., when water flow is decreasing.

### The Carriage of an IncP-1 Plasmid Has Contrasted Effects on Bacterial Adhesion

**FIGURE 2:**
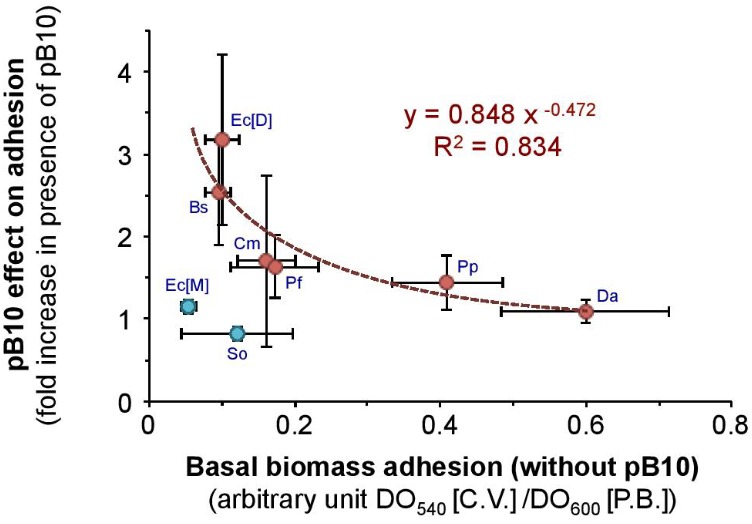
Gain in biofilm formation for pB10-bearing bacteria. Biofilm formation was compared for isogenic strains with and without the IncP-1β plasmid pB10: *B. subtilis* CM291 (Bs); *C. metallidurans* AE815 (Cm); *D. acidovorans* (Da); *E. coli* DH5α (Ec[D]), *E. coli* MG1655 (Ec[M]); *P. fluorescens* CM292 (Pf); *P. putida* SM1443 (Pp); *S. oneidensis* CM87 (So). Error bars indicate the standard error of the means (*n* = 3). The dotted line represents a regression curve related to a Spearman correlation without considering strains MG1655 and MR-1 showing that the effect of pB10 carriage on adhesion is stronger as the basal level of biofilm formation is initially low in the absence of plasmid (*r* = -0.943, *p* = 0.02). The equation of the regression curve is indicated.

Considering the selective enrichment of IncP-1 plasmid-bearing bacteria on SMs and sediments, we wondered if the mere fact of bearing this kind of MGEs was sufficient to increase cell adhesion to surfaces compared to plasmid-free bacteria. The phenotypic effect of bearing an IncP-1 plasmid was investigated using a series of isogenic bacterial strains from various species carrying or not the natural IncP-1β plasmid pB10 (Schlüter et al., 2003). Bacteria were allowed to grow as biofilm on Kaldnes polyethylene disks immerged in LB medium for 18 h, resulting in a large excess of planktonic cells. The abundance of planktonic bacteria (PB) was estimated at 600 nm (OD_600_) by spectrometry, while the abundance of the adhering biomass was measured using a classical crystal violet staining method [(CV); OD_540_] (**Table 3**). The propensity to form biofilms was finally estimated as the relative abundance of adhering cells compared to planktonic bacteria, here given in arbitrary units [(CV)/(PB)]. The results obtained (**Table 3**) showed that the carriage of pB10 significantly increased the relative amount of adhering bacteria on polyethylene surfaces (Student’s *T*-test for paired data, *p* = 5.10^-3^). Apart from the outlier strains *Escherichia coli* MG1655 and *Shewanella oneidensis* MR-1 that seem to have peculiar behaviors, the effect of pB10 carriage on adhesion is stronger when the basal level of biofilm formation is initially low in the absence of plasmid (Spearman correlation without considering strains MG1655 and MR-1: *r* = -0.943, *p* = 0.02) (**Figure 2**). It should be noted that the different adhesion behaviors observed for the different strains could not be attributed to a strain-dependent instability of the plasmid. This was estimated by plating overnight cultures grown without antibiotics on plates selective or not for pB10-bearing bacteria. Plasmid pB10 appeared to be highly stable as no significant loss could be observed in all the bacterial hosts used (Kruskal–Wallis rank sum test, *p* = 0.99), and as it had already be thoroughly assessed before for most of the bacteria and/or strains used in this work (De Gelder et al., 2007). Putting these observations back in the context of the plasmid enrichment observed on the SMs and sediments of the Orne River, it tends to demonstrate that (i) IncP-1 plasmid carriage is sufficient to promote the compartmentalization of plasmid-bearing bacteria on SMs and thus on freshly deposited sediments, and (ii) the gain in adhesion is not equally distributed among bacterial strains, which should also contribute to compartmentalize bacteria according to their nature.

**Table 3:** Effect of pB10 carriage on bacterial adhesion properties.

	Planktonic biomass (OD_600nm_)		Adhering biomass (OD_540nm_)		Relative biofilm amount (arbitrary unit: OD_540nm_/OD_600nm_)		
			
Strains/species	Without pB10	With pB10	Without pB10	With pB10	Without pB10	With pB10	Fold increase
*E. coli* DH5α	3.205 ± 0.032	3.686 ± 0.038	0.323 ± 0.072	1.101 ± 0.129	0.10 ± 0.02	0.30 ± 0.03	3.18 ± 1.04
*E. coli* MG1655	4.669 ± 0.219	4.960 ± 0.388	0.254 ± 0.047	0.309 ± 0.065	0.05 ± 0.01	0.06 ± 0.02	1.15 ± 0.09
*C. metallidurans*	3.251 ± 0.136	3.271 ± 0.313	0.530 ± 0.152	0.898 ± 0.483	0.16 ± 0.04	0.28 ± 0.16	1.70 ± 1.04
*B. subtilis*	3.988 ± 0.357	3.700 ± 0.056	0.372 ± 0.045	0.864 ± 0.222	0.10 ± 0.02	0.23 ± 0.06	2.53 ± 0.64
*P. putida*	5.787 ± 0.211	5.047 ± 0.220	2.361 ± 0.405	2.838 ± 0.092	0.41 ± 0.08	0.56 ± 0.03	1.44 ± 0.33
*P. fluorescens*	3.401 ± 1.238	2.715 ± 1.522	0.513 ± 0.047	0.591 ± 0.026	0.17 ± 0.06	0.30 ± 0.15	1.64 ± 0.38
*S. oneidensis*	4.985 ± 1.317	4.719 ± 1.142	0.503 ± 0.150	0.419 ± 0.200	0.12 ± 0.08	0.11 ± 0.08	0.82 ± 0.09
*D. acidovorans*	4.501 ± 0.488	3.634 ± 0.915	2.664 ± 0.384	2.294 ± 0.928	0.60 ± 0.12	0.67 ± 0.21	1.09 ± 0.14


*Values are expressed as a mean ± standard error (*n* = 3).*

### Adhesion to Polyethylene Disks Does Not Increase the Transfer Frequency of Plasmid pB10

**FIGURE 3:**
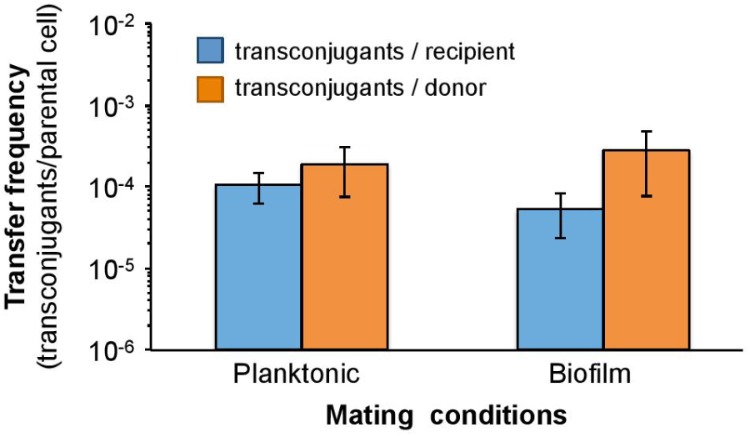
Transfer of pB10 among planktonic and polyethylene disk-adhering bacteria. Error bars indicate the standard error of the means (*n* = 4). There is not any statistical difference between the presented transfer frequencies.

Considering the fact that cell-cell contacts, required for conjugation, may be favored in biofilm and that conjugation is an intercellular mode of DNA replication, an increased frequency of transfer in biofilm may also explain enrichment of IncP-1 plasmid on SMs. This hypothesis was tested by comparing the transfer efficiency of plasmid pB10 among both planktonic bacteria and surface-associated cells. The donor bacteria *E. coli* DH5α(pB10) and the recipient strain *Cupriavidus metallidurans* AE815 were mixed and incubated for 18 h in LB medium in the presence or absence of Kaldnes polyethylene disks. Donor, recipient, and transconjugant cells were then enumerated independently from (i) the disk-free mating experiment for assessing pB10 transfer in planktonic conditions, and (ii) directly from the Kaldnes polyethylene disks for assessing pB10 transfer in biofilm. When comparing the mating conditions (planktonic versus biofilms), transfer frequencies, expressed either as transconjugant per recipient or transconjugant per donor (**Figure 3**), did not appear significantly different (Kruskal–Wallis rank sum test; *p* > 0.05). Moreover, donor, recipient, and transconjugant cells were also enumerated in the liquid medium surrounding the Kaldnes polyethylene disks. The corresponding pB10 transfer frequencies were similar to that observed for the bacteria growing as a biofilm on the disks (Wilcoxon signed rank test; *p* > 0.1). Therefore, it is unlikely that the calculation of transfer frequencies were biased by transconjugants accumulating from the liquid to the biofilm or standing out from the disks to the liquid. All in all, it thus appears that, under the conditions provided by the tests carried out on polyethylene disks, development as biofilm does not promote a higher transfer level of pB10. Therefore, between the two formulated hypotheses, the enrichment of plasmid-bearing bacteria on SMs and sediments is likely to be dominated by the adhesion properties brought by conjugative plasmids and their conjugative pili.

## Discussion

With their very close association with ARGs, MGEs undoubtedly play a central role in the dissemination of antibiotic resistance (Frost et al., 2005). Yet, demonstrating such dissemination in environmental settings remains relatively challenging as there is no easy way to evaluate the relative contribution of well-known mechanisms when combined to the complexity and heterogeneity of the environment (Elsas et al., 2002; Bellanger et al., 2014a). Occurrence-based studies have brought to light the existence of environmental reservoirs of ARGs and MGEs, suggesting the existence of environmental hotspots of ARG dissemination (Martinez, 2009; Wright, 2010; Perry and Wright, 2013). Without questioning the simple fact that ARGs can disseminate thanks to MGEs, we demonstrated with plasmid pB10 that physico-chemical interactions of bacteria with particle surfaces could preferentially promote the adhesion of IncP-1 plasmid-bearing bacteria without increasing the plasmid transfer frequency. This also means that, in the environmental context, the biofilm mode of life or adhesion to particles may also act as a “selective enrichment process” for ARGs associated with conjugative plasmids, leading to local reservoirs, even in the absence of any selective antibiotics.

The compartmentalization of IncP-1 plasmid-bearing bacteria observed in the context of the Orne River fits well the spatial clustering reported in a few metagenomic studies showing that numerous genes encoding functions associated with horizontal gene transfer and MGEs, among which type IV secretion systems (conjugation machineries), are statically overrepresented in marine bacteria attached to organo-mineral particles or algae (Burke et al., 2011; Allen et al., 2013; Ganesh et al., 2014). The authors of these works suggest that this enrichment could be due to peculiar conditions encountered in sediments and on SMs (high cell density, probable nutrient richness, shelter effect, etc.) that would promote horizontal gene transfer. In this respect, bacterial biofilms have frequently been described as hot spots of conjugative transfers (Molin and Tolker-Nielsen, 2003; Aminov, 2011; Skippington and Ragan, 2011; Madsen et al., 2012). However, to the best of our knowledge, this alternative has never been challenged experimentally by possible enrichment of plasmid-bearing bacteria due to their adhesion to mineral particles or organo-mineral aggregates.

Slight enrichment in class 1 integrons of environmental bacteria attached on the organo-mineral particles forming SMs from the Orne River by comparison to bacteria from raw waters has also been reported. To our knowledge, such a spatial clustering of class 1 integrons in an environmental compartment had never been reported. Class 1 integrons are frequently described as being hosted by IncP-1 plasmids (Popowska and Krawczyk-Balska, 2013) and as such may then show a similar compartmentalization. However, in complete genome databases, while 67% of integrons could be described as mobile (i.e., integrons associated with MGEs), only 12% of integrons (mostly class 1 integrons) appeared to be plasmid borne (Cury et al., 2016). This moderate association with plasmids could explain why class 1 integron-bearing bacteria of the Orne River do not follow the same distribution as that of the IncP-1 plasmid-bearing bacteria if the adhesion to particles was to play a central role in the compartmentalization of MGEs.

Here, we described one situation, in which the transfer of plasmid pB10 is not significantly enhanced in biofilm compared to planktonic cells. This observation is in line with a few studies showing that the transfer of conjugative plasmids in bacterial populations structured as biofilm is often limited to the donor-recipient interfaces, where bacteria are most physiologically active (Stalder and Top, 2016 and references therein). Thus, even if highly efficient in biofilm, the ability of a plasmid to transfer to the neighboring cells can appear very localized and may go unnoticed if the active recipient-donor interface represents a minute part of a well developed biofilm as it is often the case (Stalder and Top, 2016 and references therein).

With this study, we showed that IncP-1 plasmid-bearing bacteria are significantly enriched on SMs and surface sediments of the Orne River, and we provided a case study with pB10 where the carriage of plasmid can promote the adhesion of bacteria, while the adhesion to surfaces does not seem to promote significantly the transfer of the plasmid. Future efforts will focus on investigating the occurrence of class 1 integrons and IncP-1 plasmids in deeper sediment layers in order to get an insight into their abundance as a function of the local physical chemistry of the sediments, in order to determine the driving parameters associated with local MGE enrichment.

Whatever the main driving phenomenon, i.e., selective attachment of plasmid-bearing bacteria or increase in plasmid transfer in adhering bacteria, enrichment of MGEs on SMs likely means ARG/ARB enrichment as well. Considering that river SMs participate in sediment formation, this work pinpoints the role that sediments could play as ARG, ARB, and MGE reservoirs. Indeed, in the studied case presented here, SMs can be assumed as the finest fraction of sediments, predominated by clay minerals. Clay and other minerals confer to those river materials high potential to bind to organic matter and microorganisms. Thus, studying the ability of various SM physicochemical surfaces such as phyllosilicates or carbonates in being differentially colonized by ARG/MGE-free or -bearing bacteria would be of interest to predicting whether a given environment is likely to be permissive for the enrichment of MGEs and their ARGs. Ultimately, river sediments can be considered as a compartment where ARGs can accumulate and disseminate by means of MGEs to environmental and pathogenic bacteria. Numerous European rivers have been polluted, channeled, and fragmented by dams, sills, hydroelectric power stations, mills, and other obstacles. This leads to a dramatic reduction in biodiversity and water quality as well as massive disappearance of the associated wetlands, essential for the prevention of floods, and excessive sediment accumulation on riverbeds. The EU Water Framework Directive promotes river restoration and improvement of both chemical and biological water quality, notably by removing dams and other hindrances. These removal operations associated with more and more frequent hydroclimatic events and severe floods resulting from climate change will lead to remobilization of sediments and their associated content in chemical and biological pollutants such as ARGs, ARB, and MGEs. The consequences on public health of these sediment remobilizations remain an open question that should be addressed in future works/research programs.

## Author Contributions

XB, EM-P, and CM contributed substantially to the conception, the design, and the supervision of the study. MCB and HG performed experiments. MCB, HG, CM, and XB interpreted the results. XB and CM prepared the manuscript with input from the other co-authors.

## Conflict of Interest Statement

The authors declare that the research was conducted in the absence of any commercial or financial relationships that could be construed as a potential conflict of interest.
