# Supplementary material for: Suspended Materials in River Waters Differentially Enrich Class 1 Integron- and IncP-1 Plasmid-Carrying Bacteria in Sediments
Source: Front Microbiol. 2018 Jul 2;9:1443. doi: 10.3389/fmicb.2018.01443 (PMC6036612; doi:10.3389/fmicb.2018.01443)
Supplement: Supplementary file 1 [file Data_Sheet_1.DOCX]

Supplementary Material

Suspended materials in river waters differentially enrich class 1 integron- and IncP-1 plasmid-carrying bacteria in sediments

**Magali de la Cruz Barrón, Christophe Merlin, Hélène Guilloteau, Emmanuelle Montargès-Pelletier, and Xavier Bellanger ***

*** Correspondence:** Corresponding Author: [xavier.bellanger@univ-lorraine.fr](mailto:xavier.bellanger@univ-lorraine.fr)

**Supplementary Figure 1: Map of the Orne watershed presenting the geographical limits and the sampling stations.** The watershed surface area is 1276 km^2^. Beth shows the location of one dam on the Orne River. The drained surface reaches 1140 km^2^ in Beth (90%). The daily water flow of the Orne River is reported on the right plot (on the basis of data provided by Hydro Eau France, collected in Rosselange, hydrometry station). The different campaigns are indicated on the water discharge curve with vertical arrows. All the campaigns, except the last one (october 2015), were performed during flood events.

| Element | Organic carbon (%) | Si % | Al% | Fe% | Ca% | Mg% | K% | P% | Fe/Al |
| --- | --- | --- | --- | --- | --- | --- | --- | --- | --- |
| Sediment (n=8) | 4.9 ± 0.7 | 16.4 ± 1.2 | 4.2 ± 0.6 | 4.1 ± 0.4 | 11.6 ± 1.6 | 0.6 ± 0.08 | 1.0 ± 0.1 | 0.2 ± 0.04 | 1 ± 0.07 |
| SM  (n = 54) | 4.2 ± 2.1 | 16.8 ± 3.2 | 6.9 ± 2.3 | 5.3 ± 1.3 | 4.4 ± 1.6 | 0.8 ± 0.18 | 1.5 ± 0.38 | 0.3 ± 0.2 | 0.79 ± 0.08 |

**Supplementary Table 1. Element contents in Orne River suspended materials and sediments.** The number of samples is 8 for sediments and 54 for suspended materials (SMs). The element contents are rather close between surface sediments and SMs. However, SMs are richer in aluminosilicate particles, inducing higher Al, Mg and K contents, and sediments are richer in calcium carbonates inducing a higher Ca content. The contents in organic carbon are similar but SMs display a higher variability due to presence of autochthonous organic matter during the warm seasons (spring and summer).

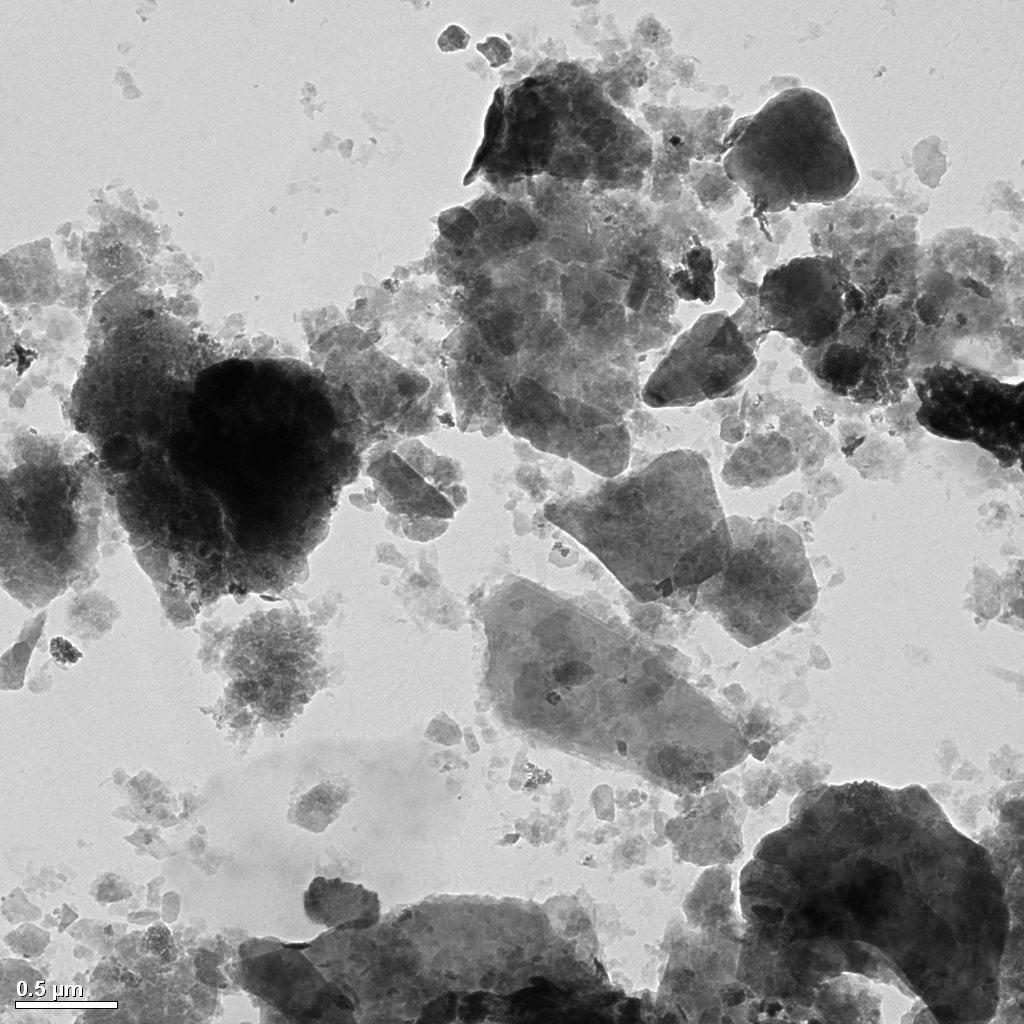


**0.5 µm**

**Supplementary Figure 2. Mineralogy of Orne River suspended materials and sediments.** On the left, a TEM picture shows clay mineral particles from suspended materials (SMs) collected in November 2014 in the Orne River. About 78 SM particles and 42 sediment particles were analyzed using the nanoprobe and the EDX detector. The EDXS analysis provided the atomic percentages of major elements for each particle (O, Mg, Al, Si, P, S, K, Ca and Fe). The graph on the right displays the results of those analysis following two atomic ratios that are representative of clay particle mineralogy. The superimposition of the sediments and SMs results strongly suggest that the clay minerals, which are the predominant constituents, have the same geological origin.

**Supplementary Figure 3.** **Particle size distribution of sediments and suspended materials.** The dotted curves correspond to suspended materials (SMs) collected during two different campaigns (low and high water flow). The grain size distribution of SMs varies as a function of climatic conditions and subsequent water flow. These two curves can be considered as the upper and lower distributions that were evidenced from river samples. SMs always appear finer than Sediments for which one representative sample is presented as a plain curve.
